# Development of anthracycline-induced dilated cardiomyopathy due to mutation on *LMNA* gene in a breast cancer patient: a case report

**DOI:** 10.1186/s12872-019-1155-7

**Published:** 2019-07-16

**Authors:** Jock Chichaco Kuruc, Armando A. Durant-Archibold, Jorge Motta, K. S. Rao, Barry Trachtenberg, Carlos Ramos, Hongyu Wang, David Gorenstein, Fredrik Vannberg, King Jordan

**Affiliations:** 1 Molecular Medicine Research Unit, Center for Biodiversity and Drug Discovery, Institute of Scientific Research and High Technology Services (INDICASAT- AIP), Panama City, Panama; 2 Acharya Nagarjuna University, Nagarjuna Nagar, India; 3 College of Natural, Exact Science and Technology, Universidad de Panama, Panama City, Panama; 4 National Secretariat for Science, Technology and Innovation, Panama City, Panama; 5 Punta Pacifica Hospital, Panama City, Panama; 6 Gorgas Memorial Institute for Health Studies, Panama City, Panama; 7 Department of Cardiology, Houston Methodist DeBakey Heart and Vascular Center, Houston, TX USA; 8 Brown Foundation Institute of Molecular Medicine, McGovern Medical School, The University of Texas Health Science Center at Houston, Houston, TX USA; 9 School of Biological Sciences, Georgia Institute of Technology, Atlanta, GA USA

**Keywords:** Cardiotoxicity, Dilated cardiomyopathy, Antrhacyclines, *LMNA* gene, Breast cancer

## Abstract

**Background:**

Anthracyclines are highly effective anticancer medication prescribed for the treatment of breast cancer. Nevertheless, the use of anthracyclines as chemotherapeutic agents involves a risk for development of cardiac toxicity which may cause restrictive and dilated cardiomyopathy. Currently, genetic predisposition is not considered as a risk factor for cardiotoxicity associated to the use of anthracyclines.

**Case presentation:**

We report the case of a 37-years old Panamanian female patient diagnosed with breast cancer who developed clinical signs of severe heart failure after treatment with doxorubicin. A diagnosis of anthracycline induced cardiomyopathy was made and treatment was initiated accordingly. A whole exome sequencing study performed to the patient showed the presence of a missense mutation in *LMNA* gene, which codifies for lamin A/C. Our results points to a correlation between the *LMNA* variant and the anthracycline cardiotoxicity developed by the woman. Improvement of the clinical symptoms and the left ventricle ejection fraction was observed after proper treatment.

**Conclusions:**

This case report suggests for the first time a potential genetic predisposition for anthracyclines induced cardiomyopathy in patients with mutations in *LMNA* gene. Perhaps chemotherapies accelerate or deliver the “second-hit” in the development of DCM in patients with genetic mutations. More data is needed to understand the contribution of *LMNA* variants that predispose to DCM in patients receiving cardiotoxic therapies.

**Electronic supplementary material:**

The online version of this article (10.1186/s12872-019-1155-7) contains supplementary material, which is available to authorized users.

## Background

Anthracyclines, such as doxorubicin and epirubicin, are highly effective and frequently used antineoplastic drugs prescribed for a variety of malignancies, including breast cancer [1, 2]. These drugs inhibit the enzyme topoisomerase II, leading to disruption in DNA replication and transcription, which in turn impedes multiplication of cancer cells. Furthermore, they promote the production of Reactive Oxygen Species (ROS) which damage proteins, DNA and cell membranes of the fastest-dividing human cancer cells [2]. Anthracycline-based chemotherapy for the treatment of breast cancer is very effective, reducing the annual mortality in women with breast cancer by 20–38%; nevertheless, the increased risk of cardiotoxicity in patients from anthracycline use has been very well described and analyzed in the medical literature [1–3]. The use of anthracyclines as chemotherapeutic agents involves an evident risk for development of cardiac toxicity generating restrictive and dilated cardiomyopathy resulting in congestive heart failure in approximately 16–20% of the treated patients [4]. The current assumptions indicate that anthracycline-induced cardiomyopathy is the result of complex multifactorial processes affecting cardiomyocytes such as inhibition of protein and nucleic acid synthesis, the generation of ROS, through interactions with topoisomerase-IIβ present in cardiomyocytes, changes in adrenergic function and adenylate cyclase, increased membrane lipid peroxidation, abnormalities in calcium ion handling, impairment of membrane binding, enzymatic activity and assembly of mitochondrial creatine kinase, induction of nitric oxide synthase enzyme, leading to nitric oxide and peroxynitrite and converse nitration/inactivation of myofibrillar creatine kinase or nitration/activation of metalloproteinases, accumulation of anthracyclines metabolites in the cardiomyocytes, and the development of apoptosis [2, 5]. The main potential risk factors described for anthracycline-induced cardiotoxicity includes cumulative and individual anthracyclines dose, age extremes, female sex, previous history of cardiovascular disease, pulmonary disease, pregnancy, infection, reduced infusion time, concomitant radiation therapy, and concomitant cardiotoxic chemotherapies (e.g her-2 anatgonists) [2]. The damage upon the heart may occur months or years after chemotherapeutic treatment at or near usual maximum doses [2, 6, 7]. Although genome-wide association investigations performed have discovered correlations between anthracycline cardiotoxicity and specific genetic mutations, there is not currently enough evidence to recommend screening patients for variants to guide clinical decision-making for cancer patients.

Here we report the case of a breast cancer patient with a mutation in *LMNA* gene who developed dilated cardiomyopathy (DCM) after treatment with doxorubicin, suggesting a potential genetic predisposition for DCM in patients with mutations in this gene. As for as our knowledge, this is the first report that correlates mutations in *LMNA* as a risk factor for the development of anthracycline-induced cardiomyopathy.

## Case presentation

**Fig. 1:**
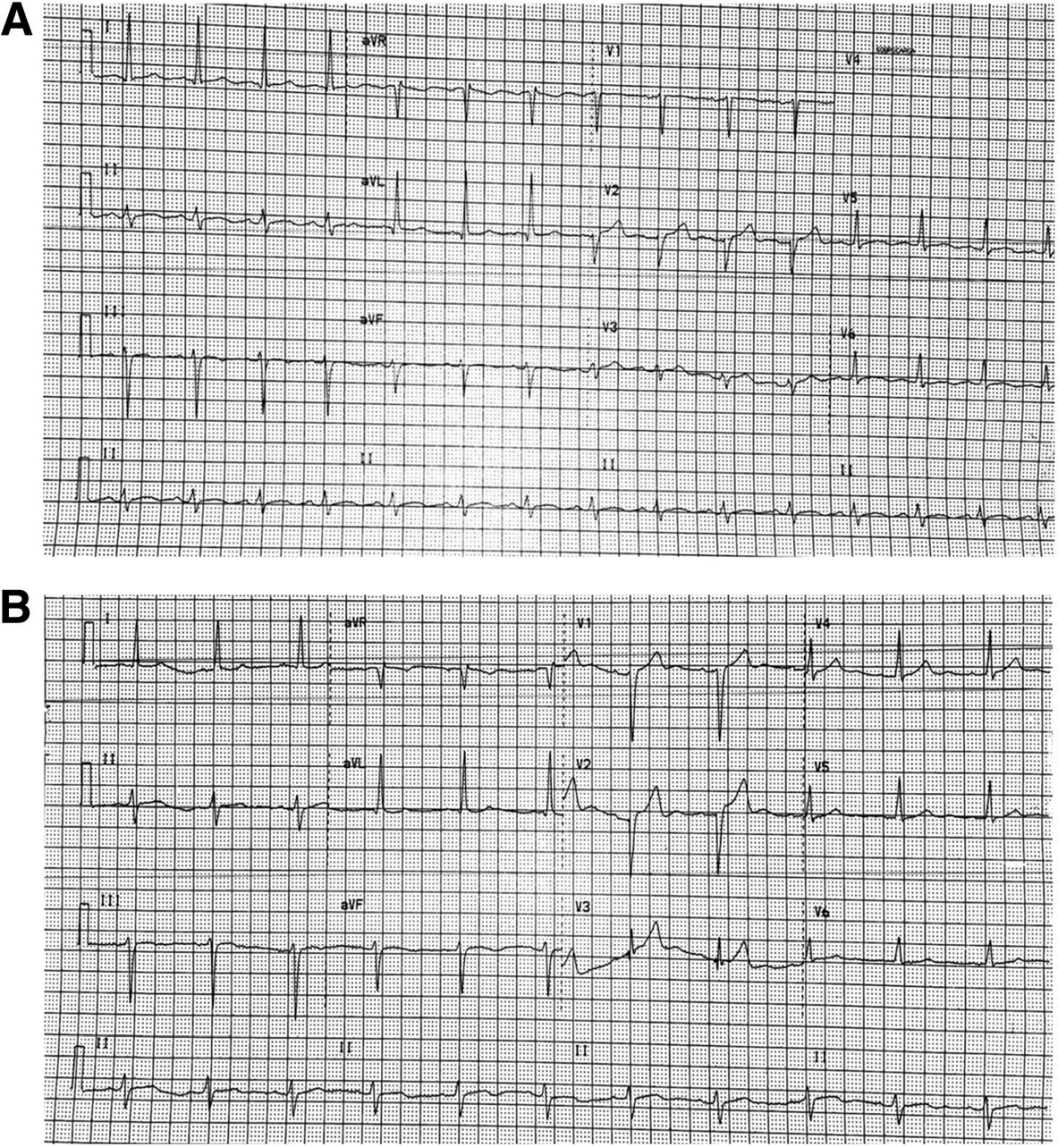
Progressive changes observed on electrocardiograms obtained from the proband. The first electrocardiogram (ECG) obtained 6 months after the beginning of the chemotherapy reported inactivable electrical zones at V1 and V3 leads with a cardiac axis of − 17°(**a**). The proband presented clinical signs of heart failure 48 months after treatment, an ECG at the time reported left anterior hemiblock and signs compatible with left cavities enlargement, cardiac axis − 46°(**b**

A 37-year-old Panamanian female patient without a pre-existing medical history, was diagnosed with a stage IIIB mammary ductal carcinoma (cT4N0M0), positive estrogen receptors (ER-positive), negative progesterone receptors (PR-negative) in her right breast in 2013, for which she received four cycles of doxorubicin (cumulative dose 240 mg/m^2^) and cyclophosphamide from January to March 2013, followed by paclitaxel from April to June. An electrocardiogram (Fig. 1) and chest radiography done prior to chemotherapy revealed no cardiac alterations. In July 2013 the patient underwent mastectomy, with subsequent radiation therapy (45 Gy in 25 fractions).

**Fig. 2:**
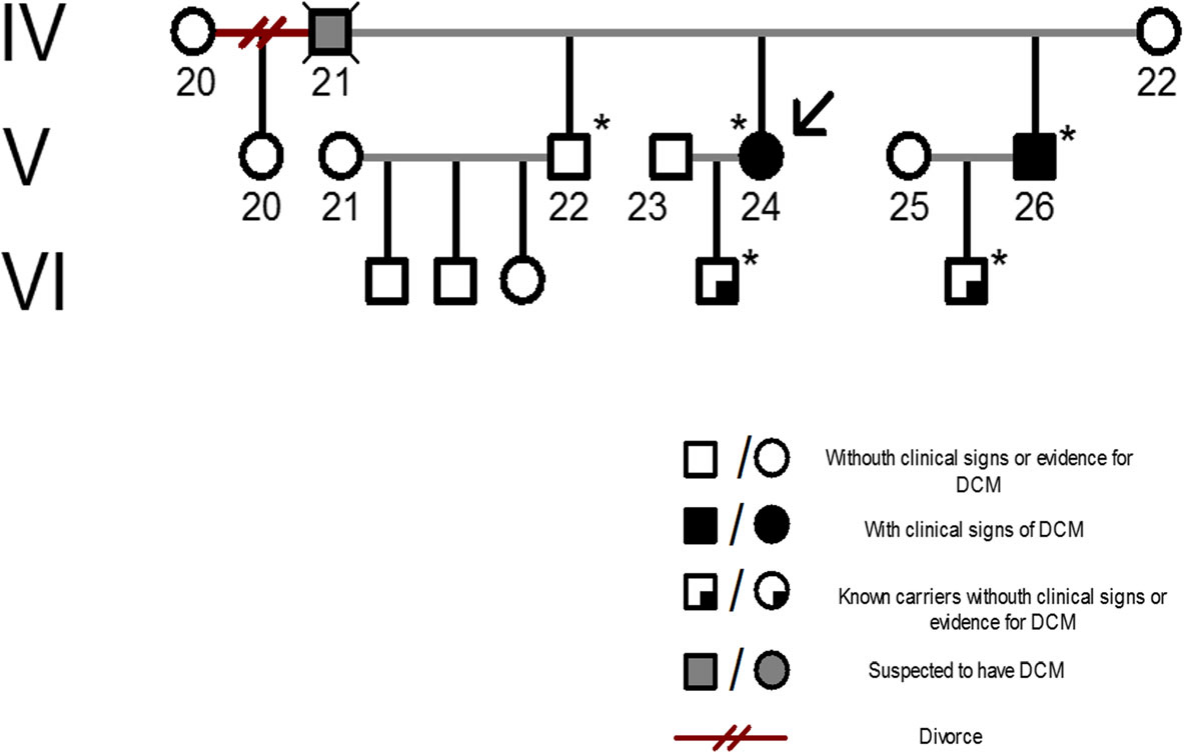
Pedigree of the proband. A summarized pedigree of the proband family is shown. Individuals are numbered according to the generation and position to which they belong. The proband (V-24) is marked by an arrow. Genomic analysis was performed in all the individuals marked with an asterisk. Circle: female; square: male; deceased individuals are marked with an X

Two years later, she was admitted to the hospital emergency department with 10 days history of orthopnea, swelling of her arms and legs, bendopnea, and fatigue. An echocardiogram was ordered, showing a reduced left ventricular systolic function (LVEF 25%). The diagnosis of anthracyclines induced cardiomyopathy was established. She was treated with a beta-blocker, diuretics and antihypertensives. After two weeks, she showed a significant improvement of her symptoms, with a LVEF of 45%. A year later, her 38-year-old brother was admitted to the hospital with a clinical presentation characterized by swelling of his legs, fatigue and minimum effort dyspnea, a left ventricular internal diameter in diastole (LVIDd) of 7.52 cm, with a LVEF of 20%. This clinical event prompted us to consider whether our patient had a genetic predisposition for DCM, and thus we performed a complete pedigree analysis of the proband (Fig. 2 and Additional file 1: Figure S1) that showed the presence of several DCM cases in the family.

In order to establish the genetic mutation responsible for the familial DCM, genomic DNA was extracted from whole-blood samples of the proband, and her brother, using a Masterpure DNA purification kit following the manufacturer’s protocol, and untargeted whole exome sequencing (WES) assessment was performed. Briefly, DNA samples were amplified prior to exome library preparation by means of GenomiPhi V3 DNA Amplification Kit. Exome libraries of the selected individuals were prepared from amplified DNA using an Illumina Nextera Rapid Capture Exome kit (version 1.1, 37 Mb). Genomic DNA was then tagmented, and amplified using polymerase chain reaction amplification (PCR); regions of interest (exons) were captured by hybridization to specific probes followed by a cycle of PCR amplification. The sequencing step was performed on an Illumina HiSeq 2500 platform, targeting for 100 bp pair-end reads, and a mean sequencing coverage average above 80x. The clinically relevant variant was subsequently confirmed by means of Sanger sequencing. The genetic investigations revealed the presence of a *LMNA*-p.Arg190Trp (NM_170707.3:c.568C > T) missense variant.

## Discussion and conclusions

We present the case of a patient with breast cancer with a mutation in the *LMNA* gene who developed cardiomyopathy after treatment with anthracycline. *LMNA* codifies for lamin A/C protein, which has a diversity of roles in the body, such as nuclear structure support, cell signaling pathway mediation, chromatin organization and DNA repair. Mutations in *LMNA* have been associated with the development of variety of pathologies such as *LMNA* cardiomyopathy [8, 9]. Variants in *LMNA* gene are responsible for around 6–8% of the reported cases of fDCM with conduction-system disease [10, 11]. The *LMNA* variant identified in the proband, *LMNA*-p.Arg190Trp (NM_170707.3:c.568C > T) which alters the helical rod domain of the protein, has been described earlier and implicated in acute types of familial DCM with, and without, conduction-system disease [12–16]. Previous case series have been reported on patients who developed anthracycline cardiomyopathy and were subsequently found to have genetic mutations known to be associated with DCM, such as *MYH7* (β-myosin heavy chain) and *TTN* (titin striated muscle protein) [17, 18]. To our knowledge, this is the first case report of a patient with anthracycline-induced cardiotioxicity because of a *LMNA* gene mutation. Despite the explosion of targeted chemotherapies and immunotherapies, the use of anthracyclines remains common as they are highly effective chemotherapies for many different types of cancers that afflict both adults and children [2, 18, 19]. As shown in our case, for cancer patients bearing the mutation highlighted in *LMNA* gene, treatment with anthracyclines can be considered a risk factor for cardiotoxicity and early development of dilated cardiomyopathy due to their genetic predisposition. More data is needed to understand the contribution and frequency of mutations (such as *LMNA*) that predispose to DCM in patients receiving potentially cardiotoxic therapies such as anthracyclines. While penetrance is incomplete in DCM genes, perhaps chemotherapies accelerate or deliver the “second-hit” in the development of DCM in patients with genetic mutations. One can envision, if further evidence supports this hypothesis, that we could truly offer precision medicine by screening patients prior to starting chemotherapy for genetic mutations that would increase their risk of developing heart failure.

## Additional file

Additional file 1: **Figure S1.** Support Information. Complete Pedigree of the proband family. Displays the complete pedigree of the proband family. Individuals are numbered according to the generation and position to which they belong. The distribution of the cases suggest the presence of an autosomal dominant disease. Circle: female; square: male; deceased individuals are marked with an X. (JPG 514 kb)

## Data Availability

All relevant data supporting this research are contained within the article.

## Abbreviations

DCM: Dilated cardiomyopathy
LVEF: Left ventricular systolic function
LVIDd: Left ventricular internal diameter in diastole
*MYH7*: β-Myosin heavy chain
PCR: Polymerase chain reaction
*TTN*: Titin striated muscle protein
WES: Whole exome sequencing
