# Supplementary figures and images for: Development of anthracycline-induced dilated cardiomyopathy due to mutation on LMNA gene in a breast cancer patient: a case report
Source: BMC Cardiovasc Disord. 2019 Jul 16;19:169. doi: 10.1186/s12872-019-1155-7 (PMC6636154; doi:10.1186/s12872-019-1155-7)

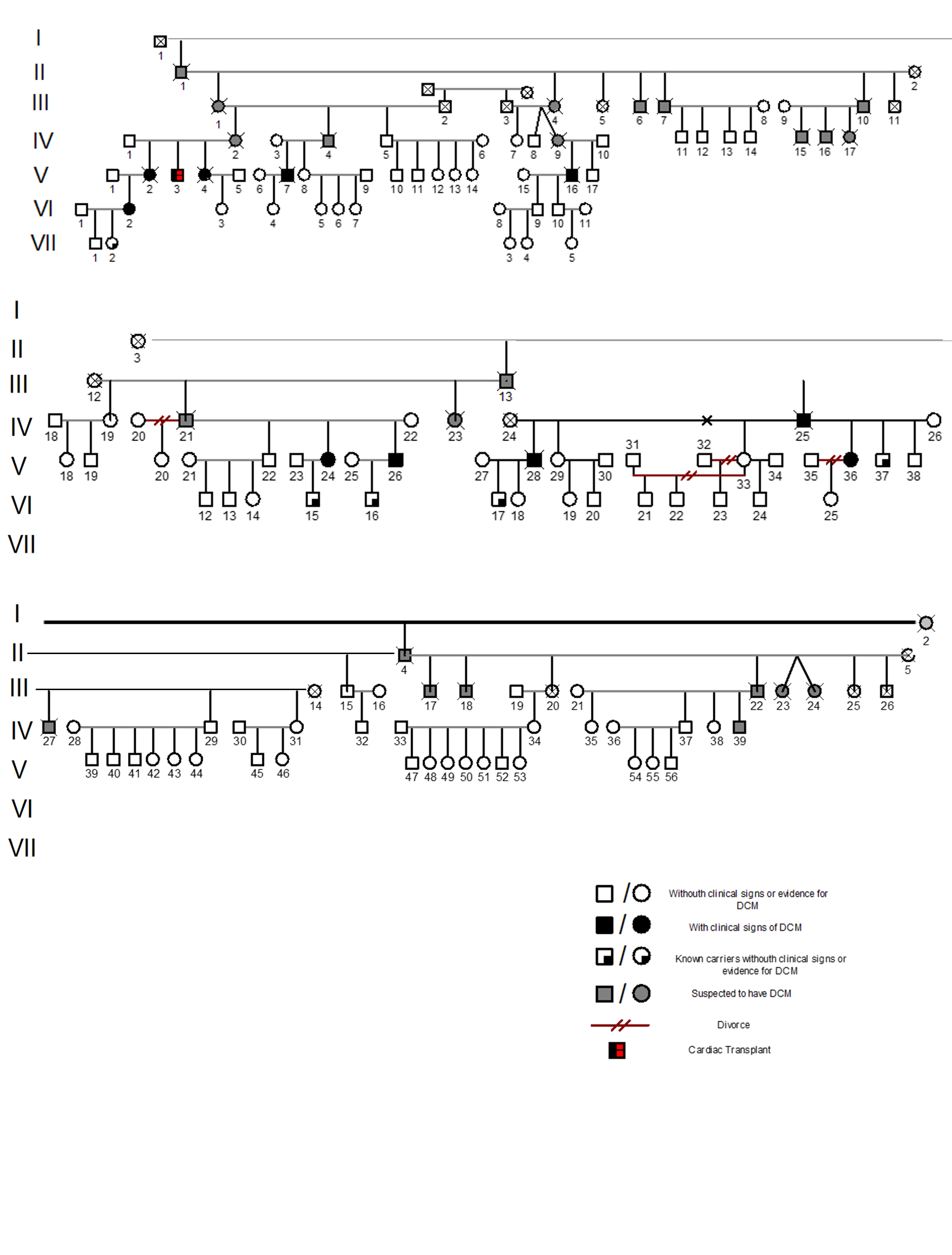

Supplement: Supplementary file 1 — Figure S1. Support Information. Complete Pedigree of the proband family. Displays the complete pedigree of the proband family. Individuals are numbered according to the generation and position to which they belong. The distribution of the cases suggest the presence of an autosomal dominant disease. Circle: female; square: male; deceased individuals are marked with an X. (JPG 514 kb) [file 12872_2019_1155_MOESM1_ESM.jpg]
